# STAT3 Hyper-IgE Syndrome—an Update and Unanswered Questions

**DOI:** 10.1007/s10875-021-01051-1

**Published:** 2021-05-01

**Authors:** Christo Tsilifis, Alexandra F. Freeman, Andrew R. Gennery

**Affiliations:** 1 Paediatric Haematopoietic Stem Cell Transplant Unit, Great North Children’s Hospital (GNCH), Royal Victoria Infirmary, Queen Victoria Road, Newcastle upon Tyne, NE1 4LP UK; 2 Translational and Clinical Research Institute, Faculty of Medical Sciences, Newcastle University, Newcastle upon Tyne, NE2 4HH UK; 3 Laboratory of Clinical Immunology and Microbiology, National Institute of Allergy and Infectious Diseases, National Institutes of Health, Bethesda, MD USA

**Keywords:** Hyper-IgE, HIES, STAT3, IgE, quality of life, vasculopathy, HSCT, Job’s syndrome

## Abstract

The hyper-IgE syndromes (HIES) are a heterogeneous group of inborn errors of immunity sharing manifestations including increased infection susceptibility, eczema, and raised serum IgE. Since the prototypical HIES description 55 years ago, areas of significant progress have included description of key disease-causing genes and differentiation into clinically distinct entities. The first two patients reported had what is now understood to be HIES from dominant-negative mutations in signal transduction and activator of transcription 3 (STAT3-HIES), conferring a broad immune defect across both innate and acquired arms, as well as defects in skeletal, connective tissue, and vascular function, causing a clinical phenotype including eczema, staphylococcal and fungal skin and pulmonary infection, scoliosis and minimal trauma fractures, and vascular tortuosity and aneurysm. Due to the constitutionally expressed nature of STAT3, initial reports at treatment with allogeneic stem cell transplantation were not positive and treatment has hinged on aggressive antimicrobial prophylaxis and treatment to prevent the development of end-organ disease such as pneumatocele. Research into the pathophysiology of STAT3-HIES has driven understanding of the interface of several signaling pathways, including the JAK-STAT pathways, interleukins 6 and 17, and the role of Th17 lymphocytes, and has been expanded by identification of phenocopies such as mutations in IL6ST and ZNF341. In this review we summarize the published literature on STAT3-HIES, present the diverse clinical manifestations of this syndrome with current management strategies, and update on the uncertain role of stem cell transplantation for this disease. We outline key unanswered questions for further study.

## Introduction

Hyper-immunoglobulin E syndrome (HIES) due to dominant-negative (DN) mutations in signal transduction and activator of transcription 3 [1, 2] (STAT3-HIES), previously Job’s syndrome, affects fewer than 1 per million population [3]. The JAK/STAT family of signal transducers comprises of four Janus kinases (JAKs) and seven STATs, collectively transducing signals from > 50 cytokines through transmembrane receptor binding and sequential phosphorylation of a JAK, then a STAT, allowing dimerization and nuclear translocation [4]. The numerous signals transduced through these pathways mean that deleterious mutations cause diverse pathology, including severe combined immunodeficiency, malignancy, autoimmunity, and myeloproliferation [4, 5].

Ligands transducing through STAT3 include IL-6, IL-10, IL-11, IL-21, IL-22, and IL-23 [5]; aberrant transduction of these pathways coupled with STAT3′s ubiquitous expression explain the multisystem manifestations of this syndrome including dermatitis, pulmonary disease, vasculopathy, and skeletal and connective tissue abnormalities. Management currently centers on prevention and treatment of infections arising from the immune deficit, which classically result from *Staphylococcus aureus* and *Candida*. Results from correcting the molecular defect in hematopoietic cells were initially discouraging; recent data, while limited, suggest that stem cell transplantation may ameliorate aspects of the syndrome.

In this review, we present an update on recent advances in the understanding of STAT3 biology, summarize the immune and extra-immune phenotypes of STAT3-HIES, describe current treatment strategies, including recent publications on the role of hematopoietic stem cell transplantation (HSCT), and outline areas for future study to advance our understanding of this syndrome.

## History of STAT3-HIES

The HIES have historically been defined by the triad of elevated IgE, dermatitis, and recurrent skin and lung infections and include diseases caused by mutations of *STAT3* [1, 2], *TYK2* [6], *PGM3* [7], *ZNF341* [8], *CARD11* [9], and *IL6ST* [10, 11]. However, recent reviews have highlighted that several non-HIES disorders also manifest with raised IgE and severe infection, while within the HIES group itself there is significant etiological, phenotypic, and immunological variation between disorders [12–14]. Notably, TYK2 deficiency does not always cause hyper-IgE [15]; PGM3 deficiency, a glycosylation defect, causes a broad phenotype that may include hyper-IgE in its spectrum [16]; and DOCK8 deficiency has been re-categorized from HIES to combined immunodeficiency [17], highlighting the challenge of grouping such heterogeneous disorders by the shared feature of raised IgE. The complex history and shifting definitions of HIES may stem from the first case report predating the discovery of IgE by some months [18] and are delineated comprehensively in other reviews [12, 14]; we focus on the syndrome first denoted as Job’s, then renamed as HIES, AD-HIES, and latterly STAT3-HIES.

**Fig. 1:**
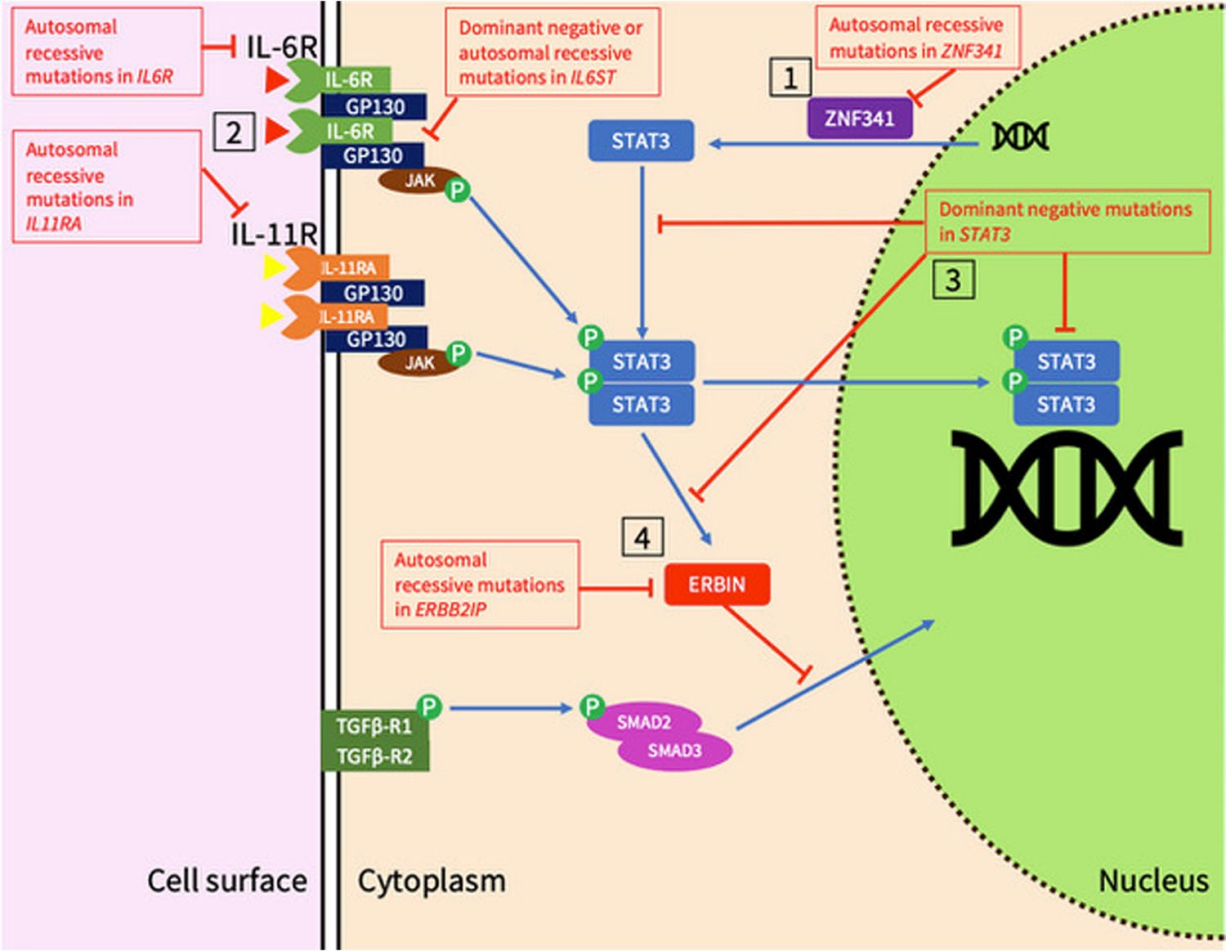
Mechanisms for disruption of STAT3-related signaling in STAT3-HIES and its phenocopies. 1. ZNF341 positively regulates STAT3 transcription [27]. 2. IL-6 and IL-11 bind to their respective receptors and form a complex with GP130, then sequentially phosphorylate first a JAK, then STAT3. IL-6 signaling may be disrupted by mutations in *IL6R*, while IL-11 signaling may be disrupted by *IL11R* mutations, and both may be affected by mutated *IL6ST* or *STAT3*. 3. Mutations in the SH2 domain of STAT3 impact tyrosine phosphorylation, while mutations in the DBD domain impact on STAT3 dimers binding to DNA [108]. 4. STAT3 activates ERBIN and disrupts TGFβ-SMAD2/3 signaling by sequestering phospho-SMAD2/3 in the cytoplasm and preventing transcriptional action of TGFβ [84]. This may be disrupted by mutations in *STAT3* or *ERBB2IP*. Key: STAT3, signal transducer and activator of transcription 3; ERBIN, ERBB2-interacting protein; ZNF341, zinc finger protein 341; GP130, glycoprotein 130

STAT3-HIES was first described in 1966, when Davis et al. described recurrent “cold” abscesses isolating *Staphylococcus aureus* in two unrelated girls sharing fair skin, eczema, and chronic sinopulmonary infection [19]. The syndrome was named after Job, the biblical figure afflicted with “sore boils from the sole of his foot unto his crown” for its distinctive and severe dermatological manifestations. The combination of recurrent skin abscess and pulmonary infection led to an initial suggestion that this may be a variant of chronic granulomatous disease [20], though subsequent bactericidal studies demonstrated normal in vitro phagocytosis of *Staphylococcus* [21]: the titular raised IgE was not identified in the index patients until 1971 [22]. Buckley et al.’s subsequent series expanded the phenotype to include chronic mucocutaneous and pulmonary fungal infection [23], impaired in vivo antibody production to novel and vaccine-strain pathogens and diminished lymphocyte stimulation by *Candida*. This was labeled the “hyper-immunoglobulin E syndrome with recurrent infections,” an entity felt to be distinct to Job’s syndrome, then still labeled a phagocytic disorder. Subsequently, Buckley and Becker demonstrated a familial link in cases, refuted the previous assertion that the syndrome affected only red-haired females, and unified the two syndromes as one entity [24]. Grimbacher et al.’s series [25] of 30 affected patients and 70 family members defined STAT3-HIES as a multi-system disorder by adding retention of primary dentition, scoliosis, and non-infectious vascular events such as cerebrovascular thromboembolic disease and aneurysms to the syndrome and identifying an autosomal dominant inheritance pattern, allowing distinction from a distinct consanguineous cohort subsequently found to have DOCK8 deficiency [26]. Identification that DN STAT3 mutations underlie the syndrome occurred through two separate groups, led by Minegishi et al. and by Holland et al. [1, 2] and has driven significant research into how disrupted STAT3 signaling generates its broad phenotype. Minegishi et al. identified heterozygous mutations in 8 patients, all located in the DNA-binding domain of STAT3, and all of which displayed loss-of-function dominant-negative effects when co-expressed with wild-type STAT3 [1]. Holland et al. identified heterozygous mutations in 50 patients, which were predicted to directly affect the DNA-binding and SRC homology 2 (SH2) domains [2]. Unlike Minegishi et al., they did not determine the loss-of-function or dominant-negative effects of the mutations. Identification of phenocopies of STAT3-HIES have aided correlation of cytokine to phenotype: biallelic mutations in ZNF341 largely phenocopy STAT3-HIES, due to the protein’s role in positively regulating STAT3 transcription [8, 27], while dominant and recessive defects in glycoprotein 130 (GP130, encoded by *IL6ST*) disturb IL-6 and IL-11 signaling, thereby causing a partial STAT3-HIES phenotype (Fig. 1) [10, 11, 28].

## Clinical Spectrum of STAT3-HIES

Patients with STAT3-HIES commonly present early, with infectious and non-infectious manifestations. Frequency of clinical manifestations from published cohorts are summarized in Table 1, while treatment recommendations, which hinge on prevention of end-organ complications by antimicrobial prophylaxis and aggressive treatment of breakthrough infections, are detailed in Table 2.

**Table 1:** Frequency of clinical manifestations of STAT3-HIES from published cohorts

	Overall	France (2012) [31]	USA (2016) [3]*	Shanghai, China (2017) [49]	Iran (2019) [119]	Italy (2019) [30]	Chongqing, China (2020) [48]	Fudan, China (2020) [120]	India (2021) [58]	Arora et al. [79]
Demographics										
Cohort size (patients)	**–**	60	85	17	19	28	20	11	27	70
Mean age at symptom onset (months)	13.9	10.5	24	12	4.8	11.5	1.5	1.9	–	–
Median age at diagnosis (years)	10.2	6.8	13.8	12	6	14.7	5.3	4.7	9.8	–
Mortality (patients)	17/235	4/60	3/83	3/17	–	1/28	3/20	–	3/27	–
Dermatological										
Dermatological symptom as first manifestation (%)	70.0	70	–	100	78	43	–	–	77.8	–
Eczema (%)	92.0	92	57.7	100	89.4	88.5	100	100	37	–
Skin abscess (%)	73.0	73	74	53	84	78	85	54.6	77.8	–
Chronic mucocutaneous candidiasis (%)	85.0	85	–	53	42	70	70	45.5	–	–
Respiratory										
Pneumonia (%)	90.0	90	72	100	84.2	60	95	–	62.9	–
Bronchiectasis (%)	65.0	65	24.7	17.7	21.1	53.4	15	–	–	–
Pneumatocele (%)	52.0	52	18.8	41.2	68.4	39.4	50	–	25.9	–
Surgical intervention (%)	22.0	22	33	23.5	–	35.7	30	18.2	–	–
Connective tissue										
Facial dysmorphism (%)	95.0	95	–	100	–	84	100	27.3	55.5	–
Retained primary teeth (%)	65.0	65	41.4	90.9	68.4	65.4	25	27.3	7.4	–
Scoliosis (%)	38.0	38	34.1	5.9	42.1	42.3	10	9.1	7.4	–
Fracture (%)	42.0	42	39	41.2	–		20	–	11.1	–
Joint hyperextensibility (%)	50.0	50	8.5	–	–	32	100	–	22.2	–
Gastrointestinal										
Gastro-esophageal reflux disease (%)	–	–	–	–	–	–	–	–	–	40
Dysphagia (%)	–	–	–	–	–	–	–	–	–	31
Gastric ulceration (%)	–	–	–	–	–	–	–	–	–	26
Intestinal strictures (%)	–	–	–	–	–	–	–	–	–	9
Other features										
Allergy (%)	48.9	–	65	35.2	42.1	~ 25	–	18.2	–	–
BCG-related complication (%)	13.4	0	–	37.5	15.7	–	38.8	–	7.4	–
Treatment										
IVIG (%)	53.0	53	30.6	–	–	–	25	72.7	13.0	–
Antibiotic prophylaxis (%)	90.0	90	> 66	–	–	–	56	100	65.2	–

Key: “Overall” represents the sum or weighted average from the cohorts listed

^*^, molecular testing for STAT3 mutation not carried out in all patients; –, not documented; *BCG*, Bacille Calmette-Guérin

**Table 2:** Summary of treatment recommendations and considerations for manifestations of STAT3-HIES

Dermatological	Recommendation	Indication/notes
Eczema	Emollients and antihistamines	Reduction of pruritus
	Monoclonal antibodies (dupilumab, anti-IL-4; omalizumab, anti-IgE)	May also reduce rates of skin abscess [121]
Staphylococcal colonization	Topical antiseptics (e.g., dilute bleach baths, swimming in pools with chlorine)	
	Anti-staphylococcal spectrum antibiotics, e.g., twice-daily co-trimoxazole [50]	Monitor for antibiotic resistance, which is seen at increased rates [122]
Mucocutaneous candidiasis	Topical antifungal treatment or daily azole antifungal prophylaxis	Warn patients of side effects such as medication interactions and photosensitivity (for voriconazole)
Pulmonary		
Recurrent pulmonary infection	Offer twice-daily co-trimoxazole prophylaxis	Pneumonia is common, and predisposes to formation of bronchiectasis and pneumatoceles [123, 124]
	Consider antifungal prophylaxis with mold-active azoles such as itraconazole in patients with parenchymal disease (bronchiectasis, pneumatocele)	*Aspergillus* confers significant mortality risk [125–127]
	CPA or ABPA may require prolonged antifungal therapy due to poor penetration into parenchymal lung disease	
	Consider immunoglobulin replacement	May reduce frequency of pneumonia, though data are limited [128]
	Offer routine immunization schedules, including live vaccinations, with the exception of the 23-valent pneumococcal polysaccharide vaccine (PPSV) Offer booster vaccinations if specific subtherapeutic IgG are observed	Avoid the 23-valent pneumococcal polysaccharide vaccine due to reports of significant local reaction, including skin necrosis [3]
	Monitor microbiological culture and sensitivities regularly	Some authors propose intravenous antibiotic therapy for *Pseudomonas* bronchiectasis exacerbations [43]
Acute infective episode	High index of suspicion for complications, e.g., empyema	Patients may lack fever or other evidence of systemic inflammation Operative management risks complications, e.g., bronchopleural fistula formation [43]
	Extend spectrum to include gram-negative bacteria (e.g., *Pseudomonas aeruginosa*) and *Aspergillus* in parenchymal disease awaiting microbiologic studies	
Parenchymal lung disease	Chest physiotherapy, airway clearance devices, and/or hypertonic saline nebulization to augment mucus clearance	May risk hemoptysis [43]
Bone and connective tissue		
Minimal trauma fractures	Optimize bone health with vitamin D supplementation	Bisphosphonates have an unclear role [72]
	Monitor bone mineral density	May not predict risk of fracture, though a reduced z-score in the distal radius may be informative [72]
Scoliosis	Monitor for development through adolescence	
Delayed exfoliation of primary dentition	Regular surveillance through childhood and adolescence, and consider removal	Consider removal to allow eruption of secondary teeth [77]
Vascular		
Coronary arterial disease	Optimize modifiable risk factors (e.g., hypertension, hyperlipidemia)	
	Consider antiplatelet agents, e.g., for primary prevention [129]	May risk hemoptysis, particularly if significant parenchymal lung disease or pulmonary arterial aneurysm is present
Other arterial aneurysms	Surveillance every 3–5 years [91]	Management of asymptomatic aneurysms is challenging, due to limited data on their natural history and the implicit risk of intervention
Reproductive health and pregnancy		
Contraception	Consider medication interactions when offering pharmacological contraception	E.g., combined oral contraceptive with azole antifungals
Pre-conception	Offer genetic counseling	
Pregnancy	Consider cessation of antimicrobial prophylaxis [130, 131]	Risk of teratogenicity
	Low threshold for presentation with pulmonary symptoms	Pregnancy may exacerbate pulmonary disease

### Bacterial Infection

Bacterial infections in STAT3-HIES are common and dominated by *Staphylococcus aureus*, affecting primarily skin and lungs, though other epithelial sites are also represented. Dermatological infection commonly begins with a newborn papulopustular rash resembling neonatal acne; this is present in 50% of patients within the first 2 weeks of life and pustulates, exudes pus, and crusts, before developing into eczema [29–31]; this rash isolates *Staphylococcus aureus* and improves with anti-staphylococcal antibiotics, distinguishing it from other neonatal pustuloses [29]. The resultant eczema is present in most patients and becomes colonized with a narrow range of *S. aureus* strains expressing higher prevalence of methicillin resistance and Panton-Valentine leucocidin [32], probably reflecting a high antibiotic burden exerting selection pressure on skin commensals.

Characteristic “cold” staphylococcal abscess formation is common and may occur in any distribution and can recur despite surgical or radiological drainage, requiring prolonged antibiotic courses [33, 34]. This diminished inflammatory response may result from impaired signaling of IL-6, a key pro-inflammatory cytokine which transduces first through its receptor, then GP130 and STAT3. Unsurprisingly, deleterious mutations in *IL6R*, *IL6ST*, and *STAT3* overlap in their clinical and immunological manifestations, reflecting IL-6′s contribution to STAT3-HIES pathogenesis [10, 11, 28, 35]. The reason for the predominance of staphylococcal infection remains unknown but likely relates to deficiency of Th17 lymphocytes, which release antimicrobial peptides and produce the IL-17 cytokine family implicated in the response to *Staphylococcus aureus*, *Candida*, and gram-negative bacteria [36–39]; their differentiation from naïve CD4 + T-lymphocytes depends on STAT3 [40]. Reduced IL-17 production may explain the predisposition to epithelial site infections: keratinocytes and bronchial epithelial cells require IL-17A for anti-staphylococcal beta-defensin secretion [41], and epithelial-site staphylococcal infection occurs in other defects disrupting the IL-17 axis including IL-17RA deficiency [42].

The lungs are the next most common site of infection in STAT3-HIES, usually infected with *Staphylococcus*, *Streptococcus pneumoniae*, or *Haemophilus influenzae* [3, 31], and pulmonary infections contribute significantly to reduced quality of life [43]. Pneumonia is seen in 80% of patients, frequently recurs, may be complicated by pleural effusion, and commonly provokes development of parenchymal disease including bronchiectasis and pneumatoceles. These provide a nidus for further colonization and subsequent re-infection with an evolving spectrum of pathogens as disease progresses, including *Pseudomonas aeruginosa* or *Aspergillus* [44]. Meanwhile, ophthalmic infections, including recurrent staphylococcal chalazia and fungal endophthalmitis, are also described [45–47].

Non-epithelial staphylococcal infection, such as liver abscess, is less common than in other defects predisposing to staphylococcal infection such as chronic granulomatous disease. Liver abscess has been described in ~ 10% and osteomyelitis and other articular infections in ~ 20% of patients [25, 31, 48, 49].

Anti-staphylococcal antibiotic prophylaxis, such as co-trimoxazole, along with antiseptic washes are recommended to prevent both dermatological and pulmonary infection [50].

### Fungal Infection

The Th17/IL-17 axis is implicated in immunity against fungi as well as bacteria, and susceptibility to mucocutaneous *Candida* infection is promoted by decreased antimicrobial peptides in the saliva of STAT3-HIES patients [51], causing an altered oral microbiome with *Candida* overgrowth. Chronic mucocutaneous infection occurs in 70% of patients, typically presenting as oral or genital thrush or onychomycosis [31].

Pulmonary fungal infection, typically by *Aspergillus*, complicates patients who develop parenchymal disease: all patients in a French cohort [31] with pulmonary *Aspergillus* had pre-existing lung damage radiologically. *Aspergillus* may manifest as chronic pulmonary aspergillosis (CPA), most frequently aspergillomas, and allergic bronchopulmonary aspergillosis (ABPA). Criteria for diagnosis of CPA involve consistent appearance on thoracic imaging, microbiological or immunological evidence of *Aspergillus*, and presence of disease for ≥ 3 months [52], and treatment may be prolonged due to poor antifungal penetration into pneumatoceles. CPA can be associated with other invasive disease [53] including of vasculature, causing life-threatening hemoptysis and contributing significantly to mortality [54]. ABPA, caused by hypersensitivity to *Aspergillus* and leading to further bronchiectasis and bronchospasm, is challenging to diagnose due to standardized criteria relying on raised IgE and eosinophilia that may be present in STAT3-HIES patients without *Aspergillus* isolation; diagnosis relies instead on classic imaging findings [55]. Infrequently, *Pneumocystis jirovecii* may cause the first episode of pneumonia. Antifungals, such as fluconazole, are useful to treat and prevent mucocutaneous *Candida*, while mold-active antifungals such as itraconazole are considered when parenchymal lung disease is present to prevent CPA and APBA, due to the high mortality associated with *Aspergillus* infection [35]. Triazoles, such as posaconazole, should be used chronically when pulmonary mold infection is present.

Endemic mycoses can cause disseminated disease: histoplasmosis frequently leads to infection of the gastro-intestinal tract and can mimic inflammatory bowel disease. *Coccidioides*, endemic in the US southwest, can cause severe meningitis and stroke, while *Cryptococcus* can cause meningitis and has been reported to cause esophageal infection [56]. Prophylaxis against these fungi should be considered for high-risk exposure, such as living in endemic areas.

### Mycobacterial Infection

Local disease may follow Bacille Calmette-Guérin (BCG) vaccination, manifesting as ipsilateral lymphadenitis and suppuration at varying incidence (7.4–39%, Table 1), though a French registry reported no adverse effects following BCG in their cohort [31]. In the few published reports of disseminated BCG, most patients did not have confirmed STAT3 mutations [57], and in endemic areas such as India tuberculosis-related disease is well-described [58], favoring BCG vaccination in high-prevalence countries. Likewise, disseminated environmental mycobacterial infection is rare, though pulmonary isolation of non-tuberculous mycobacteria (NTM), such as *Mycobacterium avium* complex and *Mycobacterium abscessus*, is found at rates comparable to cystic fibrosis (16% vs 13%) [59]; all patients with NTM isolation had bronchiectasis, suggesting that parenchymal defects predispose to this expanded spectrum of infective organisms.

### Viral Infection

Severe cutaneous viral infection occurs less commonly than in DOCK8 deficiency, though reactivation of zoster, frequently with disease limited to a single dermatome, occurs in one-third of individuals at a relatively young age; poor immunological memory to varicella may arise from impaired populations of memory CD8 + cytotoxic lymphocytes, which rely on STAT3-dependent IL-10 and IL-21 signaling [60–62]. Similarly, clearance of EBV-infected B-lymphocytes is also impaired, though evidence of translation of this to EBV-related disease is scarce, with most lymphomas being EBV-negative [63].

### Non-infectious Manifestations

#### Allergy

Despite elevated IgE, patients with STAT3-HIES demonstrate lower allergy and anaphylaxis rates compared to controls with similar IgE levels and atopic dermatitis, though still increased compared to the general population [64]. Mast cell degranulation is impaired in STAT3-HIES patients [65], and IgE formation is abnormal: it is produced in higher quantities, but to a lower affinity to allergens [66]. The exact mechanism by which hyper-IgE occurs remains unclear, though the elevated IgE levels seen in dominant and recessive *IL6ST* [10, 28] and recessive *IL6R* [35] mutations support a role for IL-6 in IgE homeostasis. STAT3-HIES patients demonstrate a skew towards IgE + class-switch recombination of memory B-lymphocytes with increased numbers compared to other immunoglobulin classes [67], possibly driven by IL-4, which acts independently of STAT3 [68]. The reduced affinity of STAT3-HIES IgE may result from impaired affinity maturation from direct switching from IgM to IgE production [67]. Common allergens include food, pollen, and drugs, with antibiotic allergy significantly more common in patients with pneumatoceles, possibly due to increased lifetime exposure [3].

#### Connective Tissue Abnormalities and Poor Wound Healing in STAT3-HIES

The connective tissue phenotype associated with STAT3-HIES varies: the characteristic facies, including a prominent forehead, deep-set eyes, broadened nasal bridge, and high-arched palate, usually develops in adolescence and may not be present in early childhood [69]. Permanent dentition develops appropriately, but there is failure of resorption of the root of primary teeth, which is necessary for eruption, and most children require primary tooth extraction [70]. Joint hyperextensibility occurs in one-third of patients, with degenerative joint disease arising in adulthood, and there is a predisposition to early-onset, minimal trauma long bone fracture. Reduced bone mineral density (BMD) is seen in 79% but does not correlate to fracture rate: while in classical osteoporosis reduced BMD is seen in the femoral neck and spine [71], in STAT3-HIES only radial BMD correlates with fracture risk [72]. IL-6 signaling through STAT3 inhibits receptor activator of NK-κB-mediated differentiation of macrophages to osteoclasts [73]; subsequently STAT3-HIES patients demonstrate a pro-resorptive state from higher number of osteoclasts than controls [74]. Interestingly, increasing BMD through treatment with anti-osteoclastogenic drugs such as bisphosphonates does not clearly reduce fracture risk, suggesting that other factors contribute [72].

Etiologically, the linkage of these defects to abnormalities in IL-6 and IL-11 signaling is supported by defects in GP130, which acts as a co-receptor for ligands including IL-6 and IL-11 upstream of STAT3 (Fig. 1), producing a syndrome that phenocopies aspects of STAT3-HIES including recurrent infection, skeletal abnormalities, and raised IgE [10]. The contribution of IL-11 to STAT3-HIES’s skeletal phenotype is supported by mutations in *IL11RA* causing an overlapping syndrome of craniofacial abnormalities without immunodeficiency [75], demonstrating how phenocopies caused by mutations along the STAT3 axis have shaped understanding of cytokine:phenotype correlation in STAT3-HIES.

An abnormal response to injury is demonstrated in several tissues, including the lung and gastrointestinal tract. As patients with STAT-HIES age, degenerative arthritis is common, likely secondary to hyperextensible joints and abnormal tissue remodeling. Joint replacements and spine stabilization surgery may be required at relatively younger ages compared to the general population [76]. A high prevalence of complications following thoracic surgery (~ 50%) is seen, the most common being bronchopleural fistula formation, which may chronically discharge and form empyemas in almost half of patients—more frequently than in surgery for similar indications in other patients [77]. Abnormal pulmonary remodeling may result from aberrant expression of matrix metalloproteinases (MMPs) -3, -8, and -9, which are dysregulated in STAT3-HIES [78]; unfortunately, there are no features to prospectively determine risk of complication.

Recent reports include other epithelial sites being affected, including intestinal perforation (both spontaneous and associated with infection, including extra-gastrointestinal infection) [79, 80]. Proposed mechanisms include gut dysbiosis from antibiotic therapy, defective IL-6 signaling supported by reports of perforation with IL-6 blockade with tocilizumab [81], and dysregulated TGF-β signaling supported by murine models of TGF-β knockout [82]. The interaction of STAT3 and TGF-β appears important given overlapping phenotypes of STAT3-HIES and Loeys-Dietz syndrome caused by *TGFBR1*/2 mutations, which manifest with connective tissue disease, raised IgE but an intact Th17 axis [83]. Indeed, IL-6 and IL-11 suppress the TGF-β pathway through STAT3 activation and recruitment of ERBIN, sequestering SMAD2/3 in the cytoplasm (Fig. 1) and preventing exertion of TGF-β’s transcriptional effects [84]: overlap in symptoms and TGF-β dysregulation between STAT3-HIES and ERBIN-deficient patients provides another indicator that IL-6 and IL-11, along with IL-17 and IL-21, are the key disease-associated cytokines in STAT3-HIES [84].

#### Vasculopathy

Vasculopathy in STAT3-HIES presents challenges to both understanding of pathogenesis and clinical management. Overall, medium-sized arterial abnormalities predominate, particularly in the coronary and intracranial vasculature; 50% of patients in a prospective study had coronary artery abnormalities with ectasia and aneurysm predominating [85] and radiographic evidence of small infarcts in some. Tortuosity and ectasia appear to develop with increasing age [86] and may be explained by STAT3-mediated transcription of VEGF and hypoxia-inducible factor (HIF)-1α, whose expression are reduced in STAT3-HIES [87], and through dysregulated MMPs: MMP-8 is specifically implicated in aneurysm formation [88]. CT coronary angiography shows subclinical atherosclerosis at similar rates to patients with coronary arterial disease, although their disease progresses to aneurysm formation, not stenosis [89]. Evidence for hypertension as a driver of arterial disease is scant; it is described in case reports and in 7/9 patients of an NIH cohort with coronary artery disease at ages 30–55 [86], but not in larger cohorts. There also appears to be an interplay between the Th17/IL-17 axis and vasculopathy in humans: murine models demonstrate aneurysm formation from IL-17A blockade [85], while blocking HIF-1α causes coronary vessel abnormalities and prevents Th17 lymphocyte differentiation [87]. However, paucity of evidence of infection in these patients and reports of progressive vascular disease after normalization of the Th17 axis and immunological indices post-HSCT [90] raise further questions about the relative contribution of immune defects in STAT3-HIES to vascular abnormalities and how these may be prevented.

Cerebrovascular aneurysms may be silent or present symptomatically with rupture, causing significant mortality [91]. Management of asymptomatic aneurysms poses a dilemma, as intervention confers significant risk. A significant proportion (86%) of patients screened with magnetic resonance imaging display focal punctate hyperintense white matter lesions, at an increased frequency compared to age-matched individuals without STAT3-HIES [85]. These are associated with arterial hypertension, smoking, silent cerebral infarcts, and vasculitides [92]; their presence in greater number and at a younger age may reflect increased ischemic injury resulting from vasculopathy. Despite this, cognitive and neuro-behavioral profiling suggests no significant difference between patients with STAT3-HIES and population norms, nor between patients with and without imaging abnormalities [92].

Intrathoracic arterial pseudoaneurysms may cause massive hemoptysis necessitating endovascular intervention [93–95]; while *Aspergillus* infection is implicated in vascular invasion and formation of pseudoaneurysms, the few reported cases and paucity of histological examination make etiological conclusions difficult to draw.

Data on primary prevention of vascular complications in STAT3-HIES are limited, and further study is required; empiric optimization of cardiovascular risk factors is recommended.

#### Obstetric and Gynecological Health

Menses may trigger exacerbations of eczema and, less commonly, pulmonary disease. Decisions surrounding contraception are challenging due to interactions with azole antifungals and increasing risk of thromboembolic disease [34]. This may be relevant given the vasculopathy these patients exhibit; alternative methods (such as progesterone-releasing intra-uterine devices) appear to be well-tolerated. Rates of miscarriage, both sporadic and recurrent, are increased; reduced STAT3 signaling in the placenta leads to insufficient proliferation of trophoblast cells and implantation failure and is associated with early miscarriage [96, 97]. Pregnancy itself may exacerbate symptoms and provides challenges in continuing antimicrobial prophylaxis due to concerns regarding teratogenicity.

#### Malignancy and Autoimmunity

Malignancy in STAT3-HIES occurs in approximately 7% of patients and is largely hematopoietic in origin, with lymphoma being most commonly described [31], contrasting with DOCK8 deficiency where malignancy is more common and includes epithelial sites such as skin [43]. There is a skew towards younger age, and B-lymphocyte non-Hodgkin subtypes, though both Hodgkin-type and T-lymphocyte lymphomas are described [98]. Treatment may be complicated by pre-existing increased infection risk compounded by chemotherapy-related myelosuppression.

STAT3-HIES patients may rarely exhibit a lupus-like phenotype associated with antinuclear and anti-double-stranded DNA antibodies [99]. This may progress to end-stage renal disease from lupus nephritis; a low threshold to investigate proteinuria or rising serum creatinine is therefore important.

## Investigations

### Genetics of STAT3

The ubiquitous expression and variety of signals transduced explain the varied phenotype of mutations in *STAT3*. Key disease-associated components of its structure include a highly-conserved SH2 domain and a DNA-binding domain (DBD), both implicated in loss-of-function and gain-of-function (GOF) syndromes. While DN STAT3 mutations cause STAT3-HIES, the phenotype of GOF mutations varies: somatic mutations are associated with large granular lymphocytic leukemia [100] and germline mutations with a variable syndrome of early-onset multiorgan autoimmunity and lymphoproliferation [101, 102]. Mutations causing STAT3-HIES and STAT3-GOF may affect the same codon [3, 103].

STAT3-DN mutations may be inherited or de novo [2] and usually occur as a result of missense or in-frame deletions [1, 50, 104], though deep intronic mutations have been described [105]. Penetrance appears complete although intra-familial phenotypes may differ, suggesting that environmental factors, such as infection history, alter the phenotype [106, 107]. Comparison between mutation site and phenotype has yielded only modest differences, with rates of non-immunological features being slightly increased in SH2 mutants [48, 106, 108]. While 118 mutations are attributed to “hyper-IgE syndrome” or “STAT3 deficiency” in the Human Gene Mutation Database, few variants had been functionally demonstrated to impair STAT3 function; however, a recent report examined all variants described to cause STAT3-HIES and found functionally that the mutations, including both in-frame and out-of-frame, were indeed dominant negative [109].

### Laboratory Analysis

Serum IgE is invariably raised, although it can decrease and even normalize over time, and eosinophilia is typical. Memory T- and B-lymphocyte populations are reduced; B-lymphocyte maturation into memory B-lymphocytes is dependent on pathways transduced through STAT3, including IL-21 and the follicular T-lymphocyte subset [110, 111], resulting in attenuated humoral responses to recall antigens but usually normal total immunoglobulin concentration. Classically, IL-17-producing Th17 lymphocytes are absent; strategies for immunophenotyping and a summary of laboratory findings are summarized in Table 3.

**Table 3:** Laboratory investigations in STAT3-HIES

Investigation	Comments
Full blood count	Eosinophilia in 70% Occasionally, anemia and/or neutropenia [3, 25]
Lymphocyte subsets	Total lymphocyte count is normal Reduced memory CD19 + CD27 + B-lymphocytes in 90% [3, 31, 132] Reduced memory T-lymphocytes [34]
Immunoglobulins	Total IgA, IgM, IgG normal Specific IgG to recall antigens is reduced Raised IgE, usually > 1000 IU/ml, which peaks in infancy and may normalize in adulthood [13]
Specialist immunophenotyping	Absent IL-17-producing Th17-lymphocytes Current strategies for identification of Th17-lymphocytes include the CD4 + CD45RA-CXCR5-CCR6 + T-lymphocyte phenotype [133, 134] or ex vivo staining for IL-17A following stimulation or induction of differentiation of naïve CD4 + T-lymphocytes [3]
Molecular analysis of *STAT3*	Heterozygous mutations are typically missense or short in-frame deletions; identification of new variants is complicated by dominant-negative and gain-of-function mutations sharing the same codon [3, 103] Any identified variant should be confirmed to be deleterious prior to attributing pathogenicity Panels may include other candidate genes for HIES, e.g., *PGM3*, *IL6ST*, and *ZNF341* (which is a recessive phenocopy of STAT3-HIES), or *DOCK8* (a combined immunodeficiency sharing features with HIES)

### NIH-HIES Score

A modified NIH-HIES score (Table 4) > 30 predicts the presence of *STAT3* mutation in patients with serum IgE > 1000 IU/ml [50]. Given the accumulation of complications as patients’ age, this score may underestimate the risk of STAT3-HIES in young children. Genetic testing to identify variants in STAT3 remains the optimal diagnostic investigation, though newly described variants should have their deleterious effect functionally validated prior to attributing pathogenicity.

**Table 4:** Revised NIH-HIES score for predicting the likelihood of a STAT3 dominant-negative mutation in a patient with serum IgE > 1000 IU/ml. Modified from Woellner et al. [50]

Clinical finding	Points						Scaling factor
	0	2	4	5	6	8	
Pneumonia (X-ray proven, total no.)	None	1	2	–	3	> 3	2.5
Newborn rash	Absent	–	Present	–	–	–	2.08
Pathological bone fractures (total no.)	None	–	1–2	–	–	> 2	3.33
Characteristic facies	Absent	Mild	–	Present	–	–	3.33
High-arched palate	Absent	Present	–	–	–	–	2.5

## Quality of Life, Natural History, and Mortality

Data on the impact on quality of life (QOL) in STAT3-HIES are limited to three series. The largest dataset, from the USIDNET registry, shows that < 25% describe no impact of their health on QOL [3], though this report was not restricted to patients with *STAT3* mutations. Fatigue and depression are common (21%) and associated with skin and pulmonary infection, as is reduced QOL, similarly to chronic granulomatous disease probands, X-linked female carriers, and X-linked agammaglobulinemia patients, demonstrating significant impact of recurrent infection and hospitalization [112–114]. A second series supports the negative impact of pulmonary symptoms on QOL, alone or in combination with dermatological disease [43]. The final dataset explores QOL and cognitive ability in 29 STAT3-HIES patients with white matter hyperintensities [92], showing a normal mean score though 20% of subjects were > 1 standard deviation below mean in physical and emotional wellbeing scores.

Data on natural history and mortality are limited, due to the few published cohorts with several sources predating molecular confirmation of STAT3-DN mutations [54]. Survival is typically into adulthood. The few series detailing cause of death skew towards younger age (range: 14 months–40 years; median: 20.5 years), primarily from pulmonary infection, particularly fungal, or complications such as pneumatocele [31, 43, 48, 49, 54]. However, with improved antifungals and expectant management, life expectancy appears to be increasing.

## The Uncertain Role of HSCT

HSCT was initially reported as unsuccessful due to progression of extra-immune features and failure to normalize serum IgE levels [115]. Following this, its role appeared more promising in a report describing HSCT as treatment of lymphoma in two patients [116], and a recent review of seven patients demonstrating satisfactory immune reconstitution and improvement in pulmonary and dermatological symptoms [99]. We recently described eight patients, including the original patient described as unsuccessful, with follow-up ranging 1–20 years and 100% survival with minimal peri-transplant complications [117]. Data from all published patients who have undergone HSCT are summarized in Table 5, and where available demonstrate improvement in rates of infection, resolution of skin disease, and stabilization or improvement of pulmonary function both clinically and radiologically. Immunologically, serum IgE fell and a normal population of IL-17-secreting Th17 lymphocytes has been demonstrated, highlighting that correcting the immune defect is both possible and beneficial to aspects of the syndrome, though the impact on non-immune manifestations such as connective tissue disease and vasculopathy is not well-understood. Notably, one patient experienced an anterior myocardial infarction associated with a coronary artery aneurysm despite normal donor chimerism and a normal Th17/IL-17 axis [118].

**Table 5:** Summary of published cases of HSCT in STAT3-HIES, with original series, transplant characteristics (age at HSCT, donor type, conditioning regimen, lymphoma as indication), presence and type of GvHD, donor chimerism at latest evaluation, and follow-up data with organ-specific status post-transplant

Series	Sex	Age at HSCT	Donor	Conditioning	Lymphoma	GvHD	Donor chimerism	Organ status post-HSCT			Survival
								Skin	Lung	Other	
Nester et al. [115]	M	46	MSD	MAC	Y	Acute	Not known	–	Interstitial pneumonitis leading to death	–	N
Goussetis et al. [118]	M	15	MSD	MAC	Y	–	Full	–	Infection-free	–	Y
	F	16	MSD	MAC	Y	–	Full	–	Infection-free	–	Y
Patel et al. [135]*	F	14	Haploidentical	RIC	–	–	Full	Abscess formation once	Infection-free	–	Y
Yanagimachi et al. [136]	F	8	MUD	RIC	–	Acute	Full	–	Recurrent aspergillosis	–	Y
	M	23	MSD	RIC	–	–	Mixed	–	Recurrent pneumatocele	–	Y
Harrison et al. [117]	M	7	MUD	RIC	–	–	Full	–	Requires nocturnal CPAP	Scoliosis, fracture	Y
	F^	7	MUD	MAC	–	Acute	Full	–	Stable appearance on CT, improved PFTs	Scoliosis, HTN	Y
	M	13	MSD	RIC	–	Acute	Full	–	Stable appearance on CT, improved PFTs	Anterior MI	Y
	M	14	MSD	RIC	–	Acute	Full	Dry, no infection	Improved appearance on CT, improved PFTs	Resolved autoimmune neutropenia	Y
	F	17	MUD	RIC	–	Acute	Full	–	Improved appearance on CT	–	Y
	M	18	MUD, MUD	RIC, RIC	–	–	Full*	–	Stable CXR changes	Septic arthritis of hip	Y
	M	13	MUD	RIC	–	–	Full	Dry, no infection	Improved symptoms	–	Y
	F	6	MSD	RIC	–	–	Full	Dry, no infection	Improved symptoms	–	Y

Key: *MSD*, matched sibling donor; *MUD*, matched unrelated donor; *MAC*, myeloablative conditioning; *RIC*: reduced intensity conditioning; *GvHD*, graft-versus-host disease; *CPAP*, continuous positive airway pressure (ventilation); *HTN*, hypertension; *PFTs*, pulmonary function tests; *MI*, myocardial infarct

^This patient was originally reported by Gennery et al. in 2000 [116]

^*^This patient had a second transplant following hyperacute rejection on D + 13

## Outstanding Questions

Recent publications have helped define the pathophysiological mechanisms underlying STAT3-HIES, particularly relating to the contributions of specific cytokines. Extant questions include the natural history of vascular anomalies, the role infection or inflammation have in their development, and how conventional risk factors impact this, in order to inform strategies for primary and secondary prevention. Further studies should explore vasculopathy in this cohort as well as other emerging symptoms, including early-onset degenerative joint and spine disease and intestinal perforation.

Understanding the impact of both infectious and non-immune manifestations on QOL would inform focus of further treatments. The role of HSCT has only begun to be explored—it would be important to know whether HSCT has any impact on any of the presumed non-immunological manifestations in this cohort. Finally, further gene discovery will likely unveil more phenocopies and add to our understanding of the complex biology of this disease.

## CME Review Questions

Question 1:

Along with STAT3 mutations, which of the following affected genes causing HIES may be inherited in a dominant manner?

A. DOCK8
B. PGM3
C. ZNF341
D. **IL6ST**
E. IL6R

Question 2:

Which of the following pathogens is not typically associated with STAT3-HIES?

A. *Staphylococcus aureus*
B. *Aspergillus fumigatus*
C. *Pseudomonas aeruginosa*
D. *Candida albicans*
E. ***Burkholderia cepacia***

Question 3:

Which of the following clinical features suggests an alternative cause of HIES?

A. Vascular abnormalities, including tortuosity
B. Retained primary dentition
C. **Severe cutaneous viral infection**
D. Abnormalities on CNS imaging
E. Bronchiectasis on cross-sectional thoracic imaging

Question 4:

Which of the following best describes the immunophenotype seen in STAT3-HIES?

A. Normal eosinophil count, normal total lymphocyte count, reduced total IgG, normal IgM
B. Normal eosinophil count, absent lymphocyte count, normal total IgG, normal IgM, presence
C. **Raised eosinophil count, normal total lymphocyte count, normal total IgG, normal IgM, absence of IL-17-producing Th17 lymphocytes**
D. Raised eosinophil count, reduced total lymphocyte count, reduced total IgG, reduced total IgM
E. Raised eosinophil count, normal total lymphocyte count, reduced total IgG, reduced total IgM, present IL-17-producing Th17 lymphocytes

Question 5:

From available evidence, which factor is most likely to impact on quality of life in patients with STAT3-HIES?

A. **Presence of pulmonary symptoms (e.g., dyspnea, reduced exercise tolerance)**
B. Presence of connective tissue symptoms (e.g., facial dysmorphism, delayed tooth eruption)
C. Presence of vascular anomalies (e.g., asymptomatic coronary artery aneurysm)
D. Presence of CNS white matter hyperintensities
E. Need for immunoglobulin replacement therapy

## Data Availability

Not applicable.
